# Markers of neuroprotection of combined EPA and DHA provided by fish oil are higher than those of EPA (*Nannochloropsis*) and DHA (*Schizochytrium*) from microalgae oils in Wistar rats

**DOI:** 10.1186/s12986-017-0218-y

**Published:** 2017-09-29

**Authors:** Paula A. Lopes, Narcisa M. Bandarra, Susana V. Martins, Joana Martinho, Cristina M. Alfaia, Marta S. Madeira, Carlos Cardoso, Cláudia Afonso, Maria C. Paulo, Rui M. A. Pinto, José L. Guil-Guerrero, José A. M. Prates

**Affiliations:** 1 CIISA, Faculdade de Medicina Veterinária, Universidade de Lisboa, Avenida da Universidade Técnica, 1300-477 Lisbon, Portugal; 2 DIVAV, Instituto Português do Mar e da Atmosfera (IPMA), Av. Brasília, 1449-006 Lisbon, Portugal; 3 CIIMAR, Universidade do Porto, Rua dos Bragas, 289, 4050-123 Porto, Portugal; 4 Depsiextracta - Tecnologias Biológicas, Lda., Quinta do Monte Novo-Taipadas, 2985-064 Canha, Portugal; 5 iMed.UL, Faculdade de Farmácia, Universidade de Lisboa, Avenida Professor Gama Pinto, 1649-003 Lisbon, Portugal; 6 Joaquim Chaves Saúde. Dr. Joaquim Chaves, Laboratório de Análises Clínicas, 1495-148 Miraflores, Algés, Portugal; 7 Departamento de Tecnología de Alimentos, Universidad de Almería, 04120 Almería, Spain

**Keywords:** Fish oil, Microalgae, Fatty acid composition, Forced swimming test, Transcriptional profile

## Abstract

**Background:**

To overcome the current overexploitation of fish rich in *n-*3 long chain polyunsaturated fatty acids (LCPUFA), microalgae have become a promising marine lipid source. The purpose of this study was to assess eicosapentaenoic acid (EPA, 20:5*n*-3) and docosahexaenoic acid (DHA, 22:6*n*-3), isolated or combined from distinct marine origins, on the promotion of neuroprotective effects.

**Methods:**

The experiment lasted for 10 weeks and involved 32 Wistar rats, divided into 4 diets (*n* = 8): a diet rich in milk fat was taken as control (Milk Fat) and compared to *n*-3 LCPUFA enriched diets, either in EPA + DHA form through fish oil (Fish Oil), or EPA through *Nannochloropsis* oil (Nanno), or DHA through *Schizochytrium* oil (Schyzo), while maintaining Milk Fat incorporation.

**Results:**

Plasma lipid profile and dopamine levels were more beneficial in Fish Oil diet. In addition, *n*-3 LCPUFA incorporation was found increased in liver and erythrocytes from Fish Oil fed rats, suggesting that fish oil is a better dietary source for fatty acids deposition in the organism than microalgae. The Forced Swimming Test revealed a positive behavioural action of EPA + DHA, in opposition to Milk Fat and Nanno diets, which had higher immobile times. mRNA levels of serotonin receptors, HT1A and HT2A along with CREB, the transmission factor for learning and memory, were higher in the hippocampus of rats fed *n*-3 LCPUFA diets comparative to Milk Fat.

**Conclusion:**

Taken together, the combination of EPA and DHA from fish oil can counteract the undesirable health effects of saturated fat based diets and benefit, in the long run, neurological function.

## Background

Ageing represents the accumulation of changes over time that are associated with or responsible for increased susceptibility to most diseases and death [1]. The European population is getting older and until 2030 the number of seniors aged over 70 is predicted to increase 40%, as will age-related neurodegenerative diseases [2, 3].

The improvement of health of the elderly population through a diet rich in essential nutrients may mitigate the effects of ageing. Precisely, essential nutrients such as *n-3* long-chain polyunsaturated fatty acids (*n*-3 LCPUFA, > 18 C), particularly eicosapentaenoic acid (EPA, 20:5*n*-3) and docosahexaenoic acid (DHA, 22:6*n*-3), are associated to a prophylactic role in certain age-related diseases with particular emphasis to some of the effects of certain degenerative diseases of the nervous system. These FA have a protective role on regulating brain development and neurotransmitter functioning and are recognised as the most beneficial FA in retarding neurological pathologies, such as Alzheimer’s, Parkinson’s and Huntington’s diseases, multiple sclerosis, schizophrenia, cognitive decline and brain ageing, major depression, acute stress and anxiety like behaviours [4–7]. Their deficiency leads to impaired neuronal function, affecting neurotransmission action [8, 9]. Accordingly, the international nutritional guidelines have recommended the need to increase EPA and DHA intake.

In the human body, DHA is synthesised in limited amounts and, therefore, must be obtained through the diet [10]. Fatty fish is the best source of *n*-3 LCPUFA [11]. However, due to the excessive and sometimes poorly regulated fishery exploitation, the depletion of worldwide fish stocks is aggravating their sustainability. Thus, search for alternative sources of *n*-3 LCPUFA to increase their availability and the consumption of these healthy FA is a major demand. Microalgae are a viable option for *n*-3 LCPUFA production because they do not require arable land for their growth and can operate as biofactories using only sunlight energy, thereby having the ability to accumulate high levels of FA under adverse environmental conditions [12]. In fact, new improvements on microalgae production have been made concerning growth optimization requirements, able to produce highly pure EPA and DHA oils [13] containing up to 30–40% of a target FA [14]. Some microalgae oils have also demonstrated safety nutritional profiles with no trace of toxicity, because they are produced under controlled conditions, thus being approved for consumption by the Food and Drug Administration.

Despite the body of evidence on *n*-3 LCPUFA positive effects on retarding and treating the neurologic conditions stated above [15, 16], a key question remains to be answered [17, 18]: are the benefits ascribed to EPA and DHA individual action or a result from the combination of both fatty acids (EPA + DHA)?

In this study, the impact of microalgae oils rich in EPA and DHA, as alternative marine lipid sources to fish oils was assessed on the neurological function of Wistar rats. We hypothesised that blue biotechnology can efficiently provide *n*-3 LCPUFA to counteract the undesirable health effects of saturated fat based diets by means of improving the biochemical profile, changing FA composition of key tissues, preventing inflammatory processes, enhancing neurological function, and modifying rat’s behaviour. To establish the effects of EPA and DHA, isolated or combined, on the promotion of neuroprotective effects, fish oil was used for comparative purposes.

## Methods

### Experimental diets

Diets were manufactured by the Experimental Diets Unit from the University of Almería, Spain. The proximate chemical composition of the diets was determined according to AOAC [19], and FA composition was assessed as described by Bandarra et al. [20]. All diets were based on the standard AIN-93 M formulation for rodents with modified lipid fractions, as follows: milk fat diet (Milk Fat group), a negative control with 20% of fat (12% from milk and 8% from soybean oil); milk fat diet plus cod liver oil (Fish Oil group), a positive control with 20% of fat (12% from milk, 4% from soybean oil and 4% from cod liver oil which is rich in EPA and DHA); milk fat diet plus *Nannochloropsis* microalga oil (Nanno group) with 20% of fat (12.5% from milk fat, 5.9% from soybean oil and 2.4% from Nanno oil which is rich in EPA); milk fat diet plus *Schizochytrium* microalga oil (Schyzo group) with 20% of fat (12.2% from milk fat, 6.5% from soybean oil and 1.8% from Schyzo oil which is rich in DHA) (Table 1). *Nannochloropsis* was purchased at Monzón BIOTECH, S.L. (Barcelona, Spain) and *Schizochytrium* was produced by Instituto Português do Mar e da Atmosfera (IPMA, Lisboa, Portugal), according to conditions outlined in previous studies [21].

**Table 1:** Ingredients, chemical composition and fatty acid profile of Milk Fat, Fish Oil, Nanno and Schyzo experimental diets

	Milk Fat	Fish Oil	Nanno	Schyzo
Ingredients (%)				
Casein	14.0	14.0	10.8	12.5
Corn starch	37.8	37.8	39.2	38.4
Maltodextrin	7.0	7.0	7.3	7.1
Sucrose	10.0	10.0	10.4	10.1
Cellulose	5.0	5.0	5.2	5.1
Soybean oil	8.0	4.0	5.9	6.5
Milk fat	12.0	12.0	12.5	12.2
Fish oil	0	4.0	0	0
Nanno oil	0	0	2.4	0
Schyzo oil	0	0	0	1.8
Cholesterol	1.25	1.25	1.30	1.27
L-cysteine	0.20	0.20	0.20	0.20
Mineral AIN-93 M Mix	3.50	3.50	3.60	3.60
Vitamin AIN-93 M Mix	1.00	1.00	1.00	1.00
Choline bitartarate	0.20	0.20	0.30	0.30
TBHQ (antioxidant)	0.001	0.001	0.001	0.001
Chemical composition (g/100 g)				
Crude protein	12.3	12.1	14.7	12.6
Crude fat	19.2	20.2	17.0	21.6
Crude fibre	3.80	3.80	3.80	3.80
Moisture	5.60	5.60	5.60	5.60
Crude ash	3.57	3.50	3.82	3.60
Carbohydrates	55.6	54.8	55.0	52.9
Total FAME (mg/g)	220	202	236	185
Energy (kcal/100 g)	444	449	432	456
Fatty acid profile (%)				
14:0	9.81	11.1	10.0	9.32
16:0	28.5	28.8	28.9	29.0
18:0	6.95	6.87	6.32	6.67
18:1*n*-9	20.3	19.1	18.0	17.0
18:2*n*-6	21.2	12.3	15.7	16.3
18:3*n*-3	2.56	1.59	1.88	2.04
18:4*n*-3	0.190	0.410	0.189	0.187
20:4*n*-6	0.086	0.138	0.587	0.110
20:5*n*-3	ND	1.15	3.16	0.10
22:6*n*-3	ND	1.19	ND	3.30
Partial sums and ratios				
Total SFA	51.1	52.9	51.2	52.1
Total MUFA	22.0	26.6	23.8	20.4
Total PUFA	24.3	17.8	21.9	23.7
Total *n*-3	2.75	4.64	5.23	5.72
Total *n*-6	21.4	12.7	16.4	17.8
*n*-3/*n*-6	0.129	0.366	0.320	0.321

*TBHQ* tertiary butyl hydroquinone, *ND* not detected

### Animals and sample collection

A total of thirty-two male Wistar rats (Harlan Interfauna Iberica SL, Barcelona, Spain), aged 28 d, were housed individually under a 12 h light-12 h dark cycle and at a temperature of 22–25 °C. After an adaptation period of 1 week to minimise stress and stabilize all metabolic conditions, rats were assigned to four body weight-matched groups with eight animals each: Milk Fat, Fish Oil, Nanno and Schyzo. Body weight and feed intake were recorded twice a week. Faeces were collected and stored at −80 °C in vacuum bags for further FA analysis. At the end of 10 weeks, rats were fasted for 12 h and killed by decapitation, under light isoflurane anaesthesia. The trunk blood was collected in lithium heparin tubes and was left to stand for 30 min. Plasma was obtained after centrifugation at 1500 *g* for 10 min. Erythrocytes were obtained after washing the pellet twice with 0·9% sodium chloride and centrifuging at 1500 *g* for 15 min and the correspondent aliquots were flash-frozen in liquid N_2_ and stored at −80 °C, for further analysis. Liver, brain and hippocampus were removed, weighed and stored at −80 °C for FA determination. Samples for gene (serotonin 5-HT1A receptor (HT1A), serotonin 5-HT2A receptor (HT2A), brain-derived neurotrophic factor (BDNF), cAMP responsive element binding protein (CREB), interleukin-6 (IL-6) and tumour necrosis factor-alpha (TNF-α) expression analysis were collected from the hippocampus, rinsed with sterile RNAse-free cold saline solution, cut into small pieces (thickness of ~0.3 cm), stabilised in RNA Later® solution (Qiagen, Hilden, Germany) and stored at −80 °C.

### Plasma biochemical assays

The plasma concentrations of total cholesterol, HDL-cholesterol, LDL-cholesterol, triacylglycerols (TAG), glucose, creatinine, urea, aspartate aminotransferase (AST), alanine aminotransferase (ALT), alkaline phosphatase (ALP) and gamma glutamyl transferase (γ-GT) were determined using standard diagnostic test kits obtained from Roche Diagnostics (Mannheim, Germany) in the Modular Hitachi Analytical System (Roche Diagnostics). The concentrations of VLDL-cholesterol and total lipids were calculated according to the Friedewald et al. [22] and Covaci et al. [23] formulas, respectively.

TNF-α and IL-6 were measured simultaneously, in duplicate, using Millipore’s MILLIPLEX rat cytokine panel (Millipore, Billerica, MA, USA). The assays were conducted according to the manufacturer’s instructions. The plate was run on a Luminex 200 Instrument using Bio-Plex Manager 4.1 standard software (Bio-Rad Laboratories, Hercules, CA, USA). Raw fluorescence data were analysed by software using a 5-parameter logistic method. The minimum detection concentrations were 2.4 pg/ml and 73.2 pg/ml for TNF-α and IL-6, respectively. The intra- and inter-assay precision of the cytokine panel were 2.3–3.6% and 11.3–14.3%, respectively.

### Fatty acid composition in faeces, liver, erythrocytes and brain

After lipid extraction by the Bligh and Dyer method [24], fatty acid methyl esters (FAME) were determined in the extracted lipids from faeces, liver, erythrocytes and brain by acid-catalysed transesterification using the methodology described by Bandarra et al. [25]. Samples were injected into a Varian Star 3800 CP gas chromatograph (Walnut Creek, CA, USA), equipped with an auto sampler with a flame ionisation detector (FID) at 250 °C. FAME were identified by comparing their retention times with those of standards (PUFA-3, Menhaden oil, and PUFA-1, Marine Source from Supelco Analytical, Sigma-Aldrich, St. Louis, MO, USA). The quantification of total FA was based on the internal standard technique, using the heneicosanoic acid (21:0) (Sigma-Aldrich). Results for each FA were expressed as a percentage of the sum of detected FA (% total FA).

### Lipid class analysis in brain

The main lipid classes were separated by analytical thin-layer chromatography (TLC) in plates coated with 0.25 mm silica gel G and developed with a mixture of chloroform:methanol:water (56:41:3 by volume), based on Bandarra et al. [25]. The developed plates were sprayed with 10% of phosphomolybdic acid in ethanol (*w*/*v*). The identification of lipid classes (polar and non-polar) was performed by comparison with standards (Sigma Chemical Co., St. Louis, MO, USA) and the quantification using a scanner and Quantity One 1-D Analysis software (version 4.5.2, Bio-Rad) [25].

### Behavioural analysis: Forced swimming test

The behavioural effects of *n*-3 LCPUFA were tested one week prior to euthanasia using the blinded Forced Swimming Test (FST), according to Porsolt et al. [26] with slight modifications. The identification of rats was ensured by a code in order to avoid bias. Each rat was put individually into vertical cylinders (60 cm height and 20 cm diameter) containing water at 25 °C (± 2 °C) with 30 cm deep for a 15 min pre-test. The cylinders were made of transparent Plexiglas as this material is able to withstand the frequent movement of the tanks and accidents better than glass. Water volume prevented the animal from escaping or touching the bottom of the cylinder. Behavioural tests were done individually, one at a time, to prevent copying and avoid bias. The room was under control to guarantee that noises, light changing or reflexes would not affect rat’s behaviour. At the end of the pre-test, the animal was taken from the water, dried with a towel to prevent hypothermia and then put back into its cage, so it could rapidly resettle to its normal conditions. The water was changed after each experiment to remove urine, faeces and odour clues left by the previous rat and thus, to avoid interferences on the next rat. 24 h later, the rat was exposed to the same aforementioned experimental conditions for a 5 min FST. This final test was recorded by a conventional video camera positioned in front of the Plexiglas cylinder. Behavioural times were calculated with proper software [27] (Ethowatcher®) and classified from most active to least active, as climbing (active upward movement), swimming (active lateral movement), floating (passive fluctuating movement) and immobile (absence of movement).

### Determination of serotonin and catecholamines

Plasma 5-hydroxytryptamine (5-HT, serotonin) and catecholamines were determined by high-performance liquid chromatography – electrochemical detection method (HPLC-ECD).

Serotonin was determined using a ClinRep kit for “serotonin in plasma/serum” (Recipe Chemicals + Instruments GmbH, Munich, Germany). Briefly, 200 μl of plasma containing N-methylserotonin as an internal standard (10 μl) was precipitated to remove proteins and lipids. The supernatant was separated on a Shimadzu series 10 HPLC system by a reversed-phase column and using an isocratic mobile phase (ClinRep, ref.: 6710) equipped with a Shimadzu series 6 electrochemical detector. The conditions for obtaining the chromatograms were as follows: a) temperature: 30 °C; b) detector: potential - 0.45 V; c) flow: 1.0 ml/min. Data obtained from the HPLC system were treated using software suitable for this type of assays (Shimadzu class 10-A software). Quality control of the system was accomplished by running ClinRep controls in two levels. The day-to-day CV was 5.4% and 5.3%, respectively. The lower limit of quantitation (LLOQ) was 1 μg/l. Catecholamines were determined using a Bio-Rad kit for “Plasma catecholamines by HPLC” (Bio-Rad, Madrid, Spain). Briefly, 50 μl of plasma containing dihydroxybenzylamine as an internal standard (5 μl) was treated with alumina (extraction phase). HPLC analysis of the supernatant was performed with an analytical cation exchange column specific for catecholamines PCAT (Bio-Rad, ref.: 195–6079) and an isocratic mobile phase purchased from Bio-Rad (ref: 195–6056). Catecholamines determinations were performed on a Shimadzu series 10 HPLC system equipped with a Shimadzu series 6 electrochemical detector. The conditions for obtaining the chromatograms were as follows: a) temperature: 30 °C; b) detector: potential - 0.50 V; c) flow: 1.1 ml/min. Data obtained from the HPLC system were treated using software suitable for this type of assays (Shimadzu class 10-A software). In each series of determinations, control samples (Lyphochek, Bio-Rad, ref. 530) were analysed in order to monitor the quality of each of the assays performed. The day-to-day CV was 4.9%. The lower limit of quantitation (LLOQ) was 25 pg/ml.

### Hippocampus RNA extraction and cDNA synthesis

Total RNA was extracted from hippocampus using the RNeasy Lipid Tissue Mini Kit (Qiagen, Hilden, Germany). To exclude possible existing genomic DNA, on-column DNase digestion with the RNase-Free DNase set (Qiagen) was performed. All procedures were based on the manufacturer’s protocol. RNA quality and concentration were determined by spectrophotometry at A260 and by the A260/A280 ratio using a NanoDrop® ND-2000c, respectively (Thermo Scientific, Wilmigton, DE, USA). To generate cDNA for qPCR, 300 ng of total RNA were reverse transcribed for 2 h at 37 °C using the High-Capacity cDNA Reverse Transcription Kit (Applied Biosystems, Foster City, CA, USA).

### Real time quantitative PCR (RT-qPCR)

mRNA expression levels were quantified using the SYBR green technology and MicroAmp Optical 96-well plates in a StepOnePlus thermocycler at standard cycling conditions (Applied Biosystems). Gene specific intron-spanning primers were designed using Primer Express® Software v.3.0 (Applied Biosystems) based on *Rattus norvegicus* sequences (www.ncbi.nlm.nih.gov). Specific sense and antisense primers used to amplify cDNA were purchased from NZYTech (Lisbon, Portugal). The specific primers were: HT1A (5′-GATCTCGCTCACTTGGCTCA-3′ and 5′-AGCGCCGAAAGTGGAGTAGA-3′); HT2A (5′-CACCACAGCCGCTTCAACTC-3′ and 5′-CACCACAGCCGCTTCAACTC-3′); BDNF (5′-AGGGATCCACACTGCCACTG-3′ and 5′-GAATTCCTCCTGCTCTGCCAG-3′); CREB (5′-CCTCCCCAGCACTTCCTACA-3′ and 5′-TCAAGCACTGCCACTCTGTTC-3′); TNF-α (5′-TCTTCTCATTCCTGCTCGTGG-3′ and 5′-GTCTGGGCCATGGAACTGAT-3′); IL-6 (5′-GGATACCACCCACAACAGACC-3′ and 5′-AGTGCATCATCGCTGTTCATACA-3′); glyceraldehyde-3-phosphate dehydrogenase (GAPDH) (5′-CAGTGCCAGCCTCGTCTCAT-3′ and 5′-CACAAGAGAAGGCAGCCCTG-3′); and ribosomal protein L27 (RPL27) (5′-GCCATGGGCAAGAAGAAGATC-3′ and 5′-GCTGGGTCTCTGAACACATCCT-3′). PCR efficiency was calculated for each amplicon using the StepOnePlus PCR System software, by amplifying 5-fold serial dilutions of pooled cDNA and running in triplicate. All primer sets were required to exhibit similar amplification efficiencies. The primer specificity and formation of primer-dimers were checked by melt curve analysis. A set of four candidate housekeeping genes was evaluated using geNorm and NormFinder, as described by Vandesompele et al. [28] and Andersen et al. [29], respectively. GADPH and RPL27 genes were selected as the most stable pair of internal controls for normalization. All analyses were performed in duplicate, and the relative amounts for each target gene were calculated using the geometric mean of housekeeping genes as normalizer. Relative expression levels were calculated as a variation of the Livak method [30] corrected for variation in amplification efficiency, as described by Fleige and Pfaffl [31].

### Statistical analysis

Statistical analysis was carried out using the Statistical Analysis System (SAS) software package, version 9.1 (SAS Institute, USA). Sample size was determined by the POWER procedure of SAS. A sample size of 8 rats provided a statistical power of at least 80% for detecting 20% differences with a 2-tailed probability of *P* < 0.05. All data were checked for normal distribution and variance homogeneity and reported as means ± standard error (SE). Data were analysed using PROC MIXED to accommodate variance heterogeneity. If significant effects were obtained, least squares means were determined using the LSMEANS option and compared using the probability difference procedure (PDIFF adjust Tukey). The PROC CORR method was used to obtain Pearson’s correlation coefficients between *n*-3/*n*-6 PUFA ratio in erythrocvtes and other parameters. *P*-values lower than 0.05 were considered statistically significant.

## Results

### Animal body composition

Body composition parameters of rats fed different *n*-3 LCPUFA sources are shown in Table 2. Most of the variables presented no changes across dietary groups, except total body weight gain that was found increased in Milk Fat in comparison to Schyzo (*P* < 0.05). Rats fed Nanno diet had higher values of daily feed intake than Fish Oil and Schyzo (*P* < 0.05). Carcass weight was found increased in Nanno relative to Schyzo (*P* < 0.05).

**Table 2:** Body composition parameters and plasma metabolites

	Milk Fat		Fish Oil		Nanno		Schyzo		Significance
	Mean	SE	Mean	SE	Mean	SE	Mean	SE	
Growth parameters and tissues weight (g)									
Final body weight	460	10.7	449	13.1	460	10.4	426	9.04	0.062
Total body weight gain	159^a^	6.59	148^ab^	7.18	152^ab^	6.05	129^b^	6.39	0.020
Daily feed intake	21.0^ab^	0.331	19.6^c^	0.330	21.8^a^	0.348	19.7^bc^	0.487	0.001
Liver	13.6	0.687	13.3	0.573	13.8	0.553	13.8	0.485	0.931
Brain	1.48	0.035	1.46	0.031	1.42	0.030	1.45	0.022	0.588
Hippocampus	0.392	0.012	0.433	0.025	0.401	0.014	0.414	0.031	0.533
*Longissimus dorsi* muscle	6.26	0.339	6.71	0.406	6.54	0.275	6.66	0.321	0.809
Epididymal fat	6.01	0.646	5.59	0.510	6.20	0.459	5.21	0.308	0.315
Retroperitoneal fat	4.62	0.654	4.05	0.405	4.63	0.320	4.54	0.413	0.712
Carcass	199^ab^	3.73	195^ab^	5.23	201^a^	3.44	185^b^	4.52	0.046
Biochemistry profile									
Total cholesterol (mg/dl)	67.3^a^	1.16	50.4^c^	1.35	54.5^c^	1.02	61.9^b^	1.41	< 0.001
HDL-Cholesterol (mg/l)	13.6	0.498	12.4	0.905	12.4	0.565	13.4	0.596	0.327
LDL-Cholesterol (mg/dl)	32.2^b^	0.631	24.0^d^	0.949	28.5^c^	0.745	35.3^a^	0.686	< 0.001
VLDL-Cholesterol (mg/dl)†	21.8^a^	0.235	14.0^b^	0.305	13.6^b^	0.371	13.2^b^	0.236	< 0.001
LDL-C/HDL-C	2.33^b^	0.045	2.01^b^	0.143	2.34^ab^	0.111	2.66^a^	0.068	< 0.001
Triacylglycerols (mg/dl)	109^a^	1.18	70.0^b^	1.52	68.1^b^	1.86	66.0^b^	1.18	< 0.001
Total lipids (mg/dl)‡	395^a^	2.75	320^c^	1.03	327^bc^	2.99	340^b^	3.66	< 0.001
Glucose (mg/dl)	174^a^	2.15	150^b^	2.04	136^c^	2.83	129^c^	3.29	< 0.001
Creatinine (mg/dl)	0.258	0.008	0.255	0.009	0.248	0.009	0.281	0.012	0.187
Urea (mg/dl)	28.5^c^	1.02	33.8^b^	1.08	32.3^bc^	1.31	41.5^a^	1.21	< 0.001
Total proteins (g/dl)	6.83	0.067	6.66	0.110	6.84	0.065	6.61	0.064	0.061
Hepatic and inflammatory markers									
AST (U/l)	112^c^	4.93	141^b^	5.26	81.3^d^	2.25	184^a^	7.08	< 0.001
ALT (U/l)	44.3^a^	2.40	50.6^a^	1.28	36.4^b^	1.49	50.5^a^	3.02	< 0.001
ALP (U/l)	94.6^b^	1.99	130^a^	6.58	85.9^c^	1.83	78.8^d^	2.47	< 0.001
γ-GT (U/l)	2.10^a^	0.118	2.19^a^	0.096	0.440^c^	0.113	1.10^b^	0.085	< 0.001
TNF-α (pg/ml)	155	11.6	168	24.7	124	6.86	141	9.88	0.080
IL-6 (pg/ml)	4219^b^	456	4420^b^	333	3772^b^	463	7760^a^	237	< 0.001

*n* = 8 per group. ^a,b,c^ Means in the same row with different superscripts are significantly different (PDIFF adjust Tukey, *P* < 0.05). †VLDL-Cholesterol = 1/5 [triacylglycerols]. ‡Total lipids = [total cholesterol] × 1.12 + [triacylglycerols] × 1.33 + 148

### Plasma metabolites and inflammatory status

Biochemistry profile, markers of hepatic and renal function, and inflammation are also shown in Table 2. Total cholesterol, VLDL-cholesterol, TAG, total lipids and glucose were higher in Milk Fat group in comparison to the others (*P* < 0.001). Rats fed Schyzo diet had increased levels of LDL-cholesterol than the others (*P* < 0.001). LDL-C/HDL-C ratio was also higher in Schyzo group relative to Milk Fat and Fish Oil (*P* < 0.001). Although creatinine remained unchanged across dietary groups (*P* > 0.05), urea was found higher in Schyzo (*P* < 0.001). Regarding the hepatic markers, AST was increased in Schyzo and decreased in Nanno (*P* < 0.001). ALT and γ-GT were reduced in Nanno (*P* < 0.001) whereas ALP was reduced in Schyzo (*P* < 0.001). TNF-α presented no variations across dietary groups (*P* > 0.05), but IL-6 was increased in Schyzo fed rats (*P* < 0.001).

### Fatty acid composition in faeces, liver and erythrocytes

FA composition in faeces is shown in Table 3. The fatty acid content was higher in Fish Oil and lower in Nanno (*P* < 0.001). Except for SFA, rats fed Nanno had increased excretion levels for MUFA (*P* < 0.001) (due to 16:1*n*-9*n*-7, 17:1 and 18:1*n*-9 (oleic acid, OA) variations), PUFA, *n*-3 PUFA (*P* < 0.001) (due to 18:3*n*-3 (α-linolenic acid, ALA) and EPA variations) and *n*-6 PUFA (*P* < 0.001) (due to 18:2*n*-6 (linoleic acid, LA) and 20:4*n*-6 (arachidonic acid, AA) variations). DHA was undetected in faeces.

**Table 3:** Total FAME (mg/g) and fatty acid composition (% total fatty acids) in faeces

	Milk Fat		Fish Oil		Nanno		Schyzo		Significance
	Mean	SE	Mean	SE	Mean	SE	Mean	SE	
Total FAME	111^ab^	17.2	126^a^	9.72	64.2^b^	4.04	108^a^	11.1	< 0.001
Fatty acids									
11:0	0.300^b^	0.064	0.540^a^	0.043	0.750^a^	0.074	0.330^b^	0.018	< 0.001
13:0	0.150^b^	0.100	0.050^b^	0.002	0.670^a^	0.042	0.060^b^	0.007	< 0.001
14:0*isobr*	0.060^b^	0.009	0.050^b^	0.004	0.320^a^	0.026	0.040^b^	0.006	< 0.001
14:0	3.93^b^	0.492	6.05^a^	0.277	5.95^a^	0.361	4.80^b^	0.201	< 0.001
15:0*isobr*	0.260^b^	0.036	0.260^b^	0.025	1.17^a^	0.045	0.250^b^	0.011	< 0.001
15:0*anteiso*	0.406	0.054	0.370	0.032	0.462	0.030	0.455	0.026	0.161
15:0	1.35^bc^	0.090	1.56^b^	0.089	1.17^c^	0.086	3.71^a^	0.112	< 0.001
16:0*anteiso*	0.140^bc^	0.018	0.100^c^	0.009	0.450^a^	0.077	0.150^b^	0.008	< 0.001
16:0	44.2^b^	1.64	46.7^b^	0.528	38.7^c^	0.804	51.7^a^	0.806	< 0.001
16:1*n*-9*n*-7	0.090^d^	0.013	0.330^b^	0.029	7.20^a^	0.418	0.170^c^	0.016	< 0.001
17:0*isobr*	0.282	0.010	0.293	0.038	0.265	0.004	0.258	0.005	0.198
17:0	0.790^b^	0.020	0.820^b^	0.014	0.640^c^	0.017	1.61^a^	0.016	< 0.001
17:1	0.200^bc^	0.017	0.220^b^	0.015	0.420^a^	0.018	0.150^c^	0.014	< 0.001
16:4*n*-3	0.067	0.010	0.062	0.006	0.058	0.013	ND	–	0.847
18:0	29.0^a^	1.50	24.5^b^	0.629	15.6^d^	0.579	21.5^c^	0.530	< 0.001
18:1*n*-9	4.89^a^	0.433	4.55^a^	0.248	4.80^a^	0.261	3.78^b^	0.136	0.002
18:1*n*-7	3.48^a^	0.335	2.86^a^	0.234	1.24^b^	0.164	2.60^a^	0.234	< 0.001
18:1*n*-5	1.20^a^	0.137	1.05^a^	0.070	0.630^b^	0.018	1.20^a^	0.124	< 0.001
19:0*isobr*	0.410^a^	0.045	0.240^b^	0.017	0.140^c^	0.006	0.210^b^	0.019	< 0.001
18:2*n*-6	1.93^b^	0.334	1.33^b^	0.060	3.16^a^	0.203	1.67^b^	0.078	< 0.001
19:0	0.140^b^	0.013	0.130^b^	0.003	0.180^a^	0.005	0.180^a^	0.004	< 0.001
18:3*n*-3	0.210^a^	0.023	0.070^c^	0.008	0.240^a^	0.021	0.120^b^	0.015	< 0.001
18:4*n*-3	0.161	0.036	0.172	0.032	0.115	0.036	0.136	0.019	0.620
20:0	1.41^a^	0.156	1.13^a^	0.093	0.790^b^	0.040	0.710^b^	0.039	< 0.001
20:1*n*-7	0.300^ab^	0.100	0.430^a^	0.037	0.110^b^	0.026	0.250^b^	0.049	< 0.001
20:2*n*-6	0.210^ab^	0.037	0.240^a^	0.014	0.110^b^	0.028	0.320^a^	0.039	< 0.001
20:4*n*-6	0.110^bc^	0.042	0.070^c^	0.009	0.820^a^	0.057	0.190^b^	0.023	< 0.001
20:5*n*-3	ND	–	0.080^b^	0.012	5.45^a^	0.439	ND	–	< 0.001
22:0	0.870^a^	0.114	0.640^a^	0.048	0.300^b^	0.034	0.400^b^	0.029	< 0.001
22:5*n*-3	0.570^a^	0.117	0.370^ab^	0.052	0.270^b^	0.014	0.510^ab^	0.114	0.014
Others	2.88^b^	0.270	4.73^b^	0.191	7.82^a^	0.301	2.54^b^	0.410	< 0.001
Partial sums and ratios									
Total SFA	83.7^ab^	1.31	83.4^b^	0.784	67.6^c^	1.38	86.4^a^	0.716	< 0.001
Total MUFA	10.2^bc^	0.821	9.44^b^	0.625	14.4^a^	0.654	8.15^c^	0.290	< 0.001
Total PUFA	3.26^b^	0.433	2.39^b^	0.126	10.2^a^	0.741	2.95^b^	0.172	< 0.001
Total *n*-3	1.01^b^	0.156	0.754^b^	0.103	6.13^a^	0.499	0.766^b^	0.125	< 0.001
Total *n*-6	2.25^bc^	0.393	1.64^c^	0.072	4.09^a^	0.258	2.18^b^	0.098	< 0.001
*n*-3/*n*-6	0.448^ab^	0.505	0.460^b^	0.075	1.50^a^	0.055	0.351^b^	0.055	< 0.001

*n* = 8 per group. ND, not detected. Total SFA = 11:0 + 13:0 + 14:0*isobr* + 14:0 + 15:0*isobr* + 15:0*anteiso* + 15:0 + 16:0*anteiso* + 16:0 + 17:0*isobr* + 17:0 + 18:0 + 19:0*isobr* + 19:0 + 20:0 + 22:0; Total MUFA = 16:1*n*-9*n*-7 + 17:1 + 18:1*n*-9 + 18:1*n*-7 + 18:1*n*-5 + 20:1*n*-7; Total PUFA = 16:4*n*-3 + 18:2*n*-6 + 18:3*n*-3 + 18:4*n*-3 + 20:2*n*-6 + 20:4*n*-6 + 20:5*n*-3 + 22:5*n*-3; Total *n*-3 = 16:4*n*-3 + 18:3*n*-3 + 18:4*n*-3 + 20:5*n*-3 + 22:5*n*-3; Total *n*-6 = 18:2*n*-6 + 20:2*n*-6 + 20:4*n*-6. ^a,b,c^ Means in the same row with different superscripts are statistically different (PDIFF adjust Tukey, *P* < 0.05)

FA composition in liver is presented in Table 4. The fatty acid content was lower in Schyzo when compared to Nanno (*P* = 0.009). SFA sum was decreased in Milk Fat fed rats relative to the others (*P* < 0.001), due to 15:0 (*P* < 0.001), 16:0 (*P* < 0.001), 17:0 (*P* < 0.001) and 18:0 (stearic acid, SA) (*P* = 0.003) variations. Rats fed Fish Oil had higher levels of total MUFA, in particular of 16:1*n*-9 (*P* = 0.008), 16:1*n*-7 (*P* < 0.001), OA (*P* < 0.001) and 20:1*n*-9 (*P* < 0.001) than Milk Fat and Schyzo (*P* < 0.001), but identical to Nanno. PUFA and n-6 PUFA were increased in Milk Fat fed rats (*P* < 0.001) in comparison to the others due to LA, 20:2*n*-6 and AA (*P* < 0.001) variations. *n*-3 sum and *n*-3/*n*-6 ratio were increased in Fish Oil relative to Milk Fat and Nanno (*P* < 0.001) due to changes in 20:4*n*-3, EPA and DHA (*P* < 0.001). While EPA reached higher values in Fish Oil and Nanno (*P* < 0.001), DHA reached higher values in Fish Oil and Schyzo (*P* < 0.001).

**Table 4:** Total FAME (mg/g) and fatty acid composition (% total fatty acids) in liver

	Milk Fat		Fish Oil		Nanno		Schyzo		Significance
	Mean	SE	Mean	SE	Mean	SE	Mean	SE	
Total FAME	317^ab^	9.38	324^ab^	13.7	340^a^	16.5	308^b^	9.09	0.009
Fatty acids									
12:0	0.103^a^	0.005	0.089^a^	0.007	0.129^a^	0.015	0.064^b^	0.005	< 0.001
14:0	1.67^b^	0.077	1.91^ab^	0.128	2.33^a^	0.137	1.19^c^	0.081	< 0.001
15:0	0.391^c^	0.009	0.525^b^	0.017	0.596^b^	0.025	1.05^a^	0.053	< 0.001
16:0	18.1^b^	0.186	24.2^a^	0.484	24.4^a^	0.510	24.3^a^	1.08	< 0.001
16:1*n*-9	0.416^b^	0.033	0.622^a^	0.055	0.421^b^	0.031	0.392^b^	0.026	0.008
16:1*n*-7	2.13^b^	0.220	3.40^a^	0.402	4.58^a^	0.471	1.74^b^	0.151	< 0.001
17:0*isobr*	0.274^b^	0.005	0.365^a^	0.019	0.357^a^	0.014	0.292^ab^	0.029	<0.001
17:0	0.221^d^	0.008	0.270^c^	0.015	0.346^b^	0.019	0.541^a^	0.025	<0.001
16:3*n*-4	0.212^b^	0.009	0.293^a^	0.020	0.322^a^	0.020	0.261^ab^	0.017	<0.001
17:1	0.089	0.005	0.109	0.008	0.098	0.007	0.104	0.015	0.192
18:0	6.10^b^	0.369	6.62^ab^	0.587	7.47^ab^	0.652	8.19^a^	0.359	0.003
18:1*n*-9	21.7^b^	0.289	24.9^a^	0.595	21.2^b^	0.329	19.2^c^	0.578	< 0.001
18:1*n*-7	2.00^a^	0.070	2.21^a^	0.060	2.25^a^	0.077	1.68^b^	0.056	< 0.001
19:0*isobr*	0.186^b^	0.006	0.227^a^	0.012	0.253^a^	0.016	0.157^b^	0.009	< 0.001
18:2*n*-6	30.7^a^	0.471	18.3^c^	0.325	21.6^b^	0.417	21.1^b^	0.259	< 0.001
19:0	0.412^a^	0.025	0.113^d^	0.005	0.167^b^	0.006	0.141^c^	0.002	< 0.001
18:3*n*-3	2.57^a^	0.293	1.33^b^	0.086	1.50^b^	0.097	1.38^b^	0.055	0.003
18:4*n*-3	0.145	0.025	0.114	0.012	0.083	0.021	0.093	0.025	0.269
20:1*n*-9	0.222^b^	0.008	0.574^a^	0.029	0.097^c^	0.003	0.071^d^	0.005	< 0.001
20:1*n*-7	0.103	0.014	0.121	0.018	0.099	0.007	0.099	0.012	0.688
20:2*n*-6	0.344^a^	0.021	0.126^b^	0.003	0.135^b^	0.004	0.100^c^	0.006	< 0.001
20:4*n*-6	5.88^a^	0.257	2.51^b^	0.308	4.37^ab^	0.254	5.40^ab^	0.324	< 0.001
20:4*n*-3	0.180^ab^	0.060	0.272^a^	0.033	0.117^b^	0.013	0.103^b^	0.014	< 0.001
20:5*n*-3	0.513^c^	0.075	2.17^a^	0.217	2.08^a^	0.209	0.900^b^	0.093	< 0.001
22:4*n*-6	0.242^a^	0.014	0.053^b^	0.003	0.091^b^	0.005	0.100^ab^	0.017	< 0.001
22:5*n*-3	0.715^b^	0.081	1.18^a^	0.070	1.31^a^	0.112	0.409^b^	0.081	< 0.001
22:6*n*-3	1.33^b^	0.067	3.85^a^	0.293	0.598^c^	0.051	6.43^a^	1.18	< 0.001
Others	3.04^ab^	0.089	3.40^b^	0.120	3.05^a^	0.057	4.53^c^	0.265	< 0.001
Partial sums and ratios									
Total SFA	27.4^b^	0.288	34.4^a^	0.846	36.1^a^	0.980	35.9^a^	1.55	< 0.001
Total MUFA	26.7^b^	0.492	32.0^a^	0.955	28.7^ab^	0.780	23.3^c^	0.756	< 0.001
Total PUFA	42.8^a^	0.378	30.2^c^	1.04	32.1^bc^	0.885	36.3^b^	1.83	< 0.001
Total *n*-3	5.45^b^	0.505	8.92^a^	0.599	5.69^b^	0.430	9.31^a^	1.36	< 0.001
Total *n*-6	37.2^a^	0.604	21.0^c^	0.555	26.1^b^	0.544	26.7^b^	0.554	< 0.001
*n*-3/*n*-6	0.148^c^	0.017	0.423^a^	0.022	0.217^b^	0.013	0.343^ab^	0.044	< 0.001

*n* = 8 per group. Total SFA = 12:0 + 14:0 + 15:0 + 16:0 + 17:0*isobr* + 17:0 + 18:0 + 19:0*isobr* + 19:0; Total MUFA = 16:1*n*-9 + 16:1*n*-7 + 18:1*n*-9 + 18:1*n*-7 + 20:1*n*-9 + 20:1*n*-7; Total PUFA = 16:3*n*-4 + 18:2*n*-6 + 18:3*n*-3 + 18:4*n*-3 + 20:2*n*-6 + 20:4*n*-6 + 20:4*n*-3 + 20:5*n*-3 + 22:4*n*-6 + 22:5*n*-3 + 22:6*n*-3; Total *n*-3 = 18:3*n*-3 + 18:4*n*-3 + 20:4*n*-3 + 20:5*n*-3 + 22:5*n*-3 + 22:6*n*-3; Total *n*-6 = 18:2*n*-6 + 20:2*n*-6 + 20:4*n*-6 + 22:4*n*-6. ^a,b,c^ Means in the same row with different superscripts are statistically different (PDIFF adjust Tukey*, P* < 0.05)

FA composition in erythrocytes is shown in Table 5. It exhibited several resemblances to liver’s, in particular for MUFA (*P* < 0.001), *n*-3 PUFA (*P* < 0.001) and *n*-3/*n*-6 ratio (*P* < 0.001). The same applies for EPA (*P* < 0.001) and DHA (*P* < 0.001). The *n*-6 PUFA reached the highest value in Milk Fat fed rats in comparison to the others (*P* < 0.001), due to variations in LA (*P* < 0.001), 20:2*n*-6 (*P* < 0.001), AA (*P* < 0.001) and 22:4*n*-6 (*P* < 0.001). The SFA sum was similar across diets (*P* > 0.05), although small changes were observed for 15:0, 17:0, 18:0, 19:0, 20:0 and 22:0 (*P* < 0.001).

**Table 5:** Total FAME (mg/g) and fatty acid composition (% total fatty acids) in erythrocytes

	Milk Fat		Fish Oil		Nanno		Schyzo		Significance
	Mean	SE	Mean	SE	Mean	SE	Mean	SE	
Total FAME	9.82	0.718	10.6	0.455	10.8	0.320	11.7	0.889	0.521
Fatty acids									
14:0	0.550	0.052	0.680	0.044	0.730	0.037	0.780	0.170	0.068
15:0	0.360^a^	0.024	0.430^a^	0.027	0.450^b^	0.011	0.940	0.066	< 0.001
16:0*anteiso*	2.34	0.071	2.31	0.062	2.61	0.094	2.24	0.111	0.050
16:0	23.9	1.18	25.7	1.09	26.0	0.459	24.8	0.942	0.329
16:1*n*-9	0.120^b^	0.017	0.190^a^	0.007	0.160^b^	0.005	0.160^ab^	0.012	0.005
16:1*n*-7	0.320^c^	0.033	0.520^ab^	0.035	0.720^a^	0.068	0.420^bc^	0.040	< 0.001
17:0*isobr*	0.260^c^	0.006	0.270^c^	0.007	0.300^b^	0.004	0.330^a^	0.005	< 0.001
17:0	0.460^c^	0.010	0.440^c^	0.010	0.580^b^	0.007	0.760^a^	0.009	< 0.001
16:3*n*-4	0.300^b^	0.022	0.320^ab^	0.029	0.350^ab^	0.030	0.390^a^	0.016	0.009
17:1	0.230^a^	0.012	0.210^a^	0.012	0.190^a^	0.015	0.150^b^	0.008	< 0.001
16:3*n*-3	2.44	0.066	2.42	0.077	2.50	0.065	2.64	0.162	0.609
16:4*n*-3	1.27^a^	0.034	1.16^ab^	0.038	1.08^b^	0.035	0.890^c^	0.042	< 0.001
18:0	13.8^a^	0.138	13.1^b^	0.204	13.5^ab^	0.213	12.9^b^	0.228	0.004
18:1*n*-9	7.84^b^	0.073	8.50^a^	0.058	8.00^ab^	0.257	8.04^ab^	0.281	< 0.001
18:1*n*-7	2.24^a^	0.067	2.29^a^	0.051	2.21^a^	0.048	1.92^b^	0.047	< 0.001
18:2*n*-6	13.2^a^	0.130	11.6^b^	0.195	11.1^b^	0.172	11.2^b^	0.424	< 0.001
19:0	0.090^a^	0.009	0.070^b^	0.002	0.080^a^	0.002	0.080^a^	0.005	< 0.001
18:3*n*-3	0.140^a^	0.019	0.090^b^	0.004	0.060^c^	0.005	0.070^bc^	0.011	< 0.001
20:0	0.110^ab^	0.006	0.090^b^	0.006	0.100^ab^	0.005	0.130^a^	0.013	0.047
20:1*n*-9	0.080^b^	0.005	0.400^a^	0.043	0.070^b^	0.009	0.090^b^	0.012	< 0.001
20:1*n*-7	0.077	0.006	0.094	0.007	0.086	0.009	0.085	0.004	0.339
20:2*n*-6	0.370^a^	0.023	0.200^b^	0.024	0.170^b^	0.009	0.190^b^	0.016	< 0.001
20:4*n*-6	20.5^a^	0.617	14.6^c^	0.570	17.7^b^	0.172	17.9^b^	0.725	< 0.001
20:4*n*-3	0.410^ab^	0.386	0.080^b^	0.012	0.060^b^	0.002	0.490^a^	0.110	< 0.001
20:5*n*-3	0.260^c^	0.026	3.08^a^	0.114	2.33^b^	0.054	0.470^c^	0.254	< 0.001
22:0	0.290^b^	0.026	0.270^b^	0.032	0.280^b^	0.009	0.390^a^	0.354	0.002
22:4*n*-6	1.51^a^	0.118	0.250^d^	0.023	0.550^b^	0.021	0.390^c^	0.281	< 0.001
22:5*n*-6	0.340^b^	0.021	0.100^d^	0.007	0.140^c^	0.008	1.75^a^	1.50	< 0.001
22:5*n*-3	1.94^bc^	0.426	2.55^b^	0.177	3.50^a^	0.094	0.950^c^	0.654	< 0.001
24:0	0.890	0.089	0.827	0.077	0.849	0.038	0.932	0.625	0.747
22:6*n*-3	1.22^c^	0.097	4.24^b^	0.353	0.680^d^	0.061	5.78^a^	0.484	< 0.001
24:1*n*-9	0.452	0.046	0.570	0.070	0.400	0.032	ND	–	0.106
Others	1.69^b^	0.029	2.37^a^	0.080	2.47^a^	0.026	1.76^b^	0.057	< 0.001
Partial sums and ratios									
Total SFA	43.0	1.16	44.2	1.03	45.5	0.646	44.3	0.896	0.221
Total MUFA	11.4^b^	0.119	12.8^a^	0.148	11.8^ab^	0.396	10.9^b^	0.332	< 0.001
Total PUFA	43.9^a^	1.14	40.7^ab^	1.11	40.2^b^	0.389	43.1^a^	1.00	0.008
Total *n*-3	7.68^c^	0.701	13.6^a^	0.691	10.2^b^	0.155	11.3^a^	0.556	< 0.001
Total *n*-6	35.9^a^	0.659	26.8^d^	0.457	29.7^c^	0.166	31.4^b^	0.576	< 0.001
*n*-3/*n*-6	0.214^c^	0.018	0.509^a^	0.018	0.344^b^	0.004	0.359^b^	0.015	< 0.001

*n* = 8 per group. ND, not detected. Total SFA = 14:0 + 15:0 + 16:0*anteiso* + 16:0 + 17:0*isobr* + 17:0 + 18:0 + 19:0 + 20:0 + 22:0 + 24:0; Total MUFA = 16:1*n*-9 + 16:1*n*-7 + 17:1 + 18:1*n*-9 + 18:1*n*-7 + 20:1*n*-9 + 20:1*n*-7 + 24:1*n*-9; Total PUFA = 16:3*n*-4 + 16:3*n*-3 + 16:4*n*-3 + 18:2*n*-6 + 18:3*n*-3 + 20:2*n*-6 + 20:4*n*-6 + 20:4*n*-3 + 20:5*n*-3 + 22:4*n*-6 + 22:5*n*-6 + 22:5*n*-3 + 22:6*n*-3; Total *n*-3 = 16:3*n*-3 + 16:4*n*-3 + 18:3*n*-3 + 20:4*n*-3 + 20:5*n*-3 + 22:5*n*-3 + 22:6*n*-3; Total *n*-6 = 18:2*n*-6 + 20:2*n*-6 + 20:4*n*-6 + 22:4*n*-6 + 22:5*n*-6. ^a,b,c^ Means in the same row with different superscripts are statistically different (PDIFF adjust Tukey*, P* < 0.05)

### Fatty acid composition and major phospholipid classes in brain

FA composition in brain is presented in Table 6. Whereas the liver displayed the highest number of variations depending on diet, the brain tissue was less responsive. SFA, MUFA, PUFA and *n*-3 PUFA were unchanged across dietary treatments (*P* > 0.05). *n*-6 PUFA was higher in Milk Fat in comparison to Fish Oil (*P* = 0.018), but identical to Nanno and Schyzo, most at the expenses of LA (*P* = 0.011) and 22:4*n*-6 (*P* = 0.031). The *n*-3/*n*-6 ratio reached the highest value in Fish Oil fed rats relative to Milk Fat and Nanno (*P* = 0.000). As expected, EPA was not detected in Milk Fat and it was best incorporated in Fish Oil fed rats (*P* = 0.000). DHA did not vary across diets (*P* > 0.05).

**Table 6:** Total FAME (mg/g), fatty acid composition (% total fatty acids) and major phospholipid classes in brain

	Milk Fat		Fish Oil		Nanno		Schyzo		Significance
	Mean	SE	Mean	SE	Mean	SE	Mean	SE	
Total FAME	153	8.04	175	12.1	169	8.08	157	5.01	0.354
Fatty acids									
14:0	0.172	0.010	0.156	0.021	0.147	0.016	0.144	0.013	0.365
16:0*anteiso*	2.23	0.101	2.14	0.089	2.36	0.180	2.13	0.863	0.641
16:0	18.9	0.819	18.6	1.42	18.9	1.26	17.6	0.011	0.692
16:1*n*-9	0.112	0.006	0.120	0.008	0.114	0.007	0.093	0.021	0.293
16:1*n*-7	0.380	0.019	0.444	0.039	0.407	0.025	0.379	0.021	0.406
16:3*n*-4	0.239	0.024	0.284	0.025	0.217	0.028	0.246	0.033	0.348
16:3*n*-3	3.90	0.055	4.10	0.086	4.36	0.259	4.03	0.057	0.095
16:4*n*-3	1.45	0.119	1.40	0.066	1.44	0.057	1.42	0.086	0.963
18:0	18.9	0.382	19.6	0.181	19.9	0.332	19.2	0.283	0.174
18:1*n*-9	15.8	0.427	16.6	0.277	15.9	0.246	16.1	0.483	0.238
18:1*n*-7	3.03	0.095	2.93	0.041	3.01	0.031	2.88	0.085	0.239
18:2*n*-6	1.04^a^	0.064	0.920^ab^	0.038	0.810^b^	0.028	0.830^ab^	0.082	0.011
19:0	0.047	0.005	0.051	0.002	0.052	0.004	0.055	0.002	0.420
18:4*n*-3	0.051	0.004	0.045	0.008	0.059	0.006	0.052	0.002	0.569
20:0	0.459	0.069	0.437	0.036	0.443	0.035	0.442	0.038	0.994
20:1*n*-9	1.44	0.286	1.33	0.144	1.36	0.103	1.44	0.172	0.962
20:1*n*-7	0.449	0.077	0.398	0.037	0.413	0.030	0.430	0.044	0.915
20:2*n*-6	0.151	0.019	0.110	0.008	0.120	0.007	0.097	0.008	0.050
20:4*n*-6	10.1	0.284	9.28	0.209	9.60	0.476	9.59	0.181	0.202
20:5*n*-3	ND	–	0.070^a^	0.006	0.040^b^	0.004	0.020^c^	0.003	0.000
22:0	0.478	0.074	0.407	0.049	0.429	0.055	0.456	0.051	0.844
22:1*n*-11	0.221	0.045	0.176	0.024	0.197	0.019	0.199	0.031	0.810
22:1*n*-9	0.101	0.004	0.113	0.011	0.115	0.015	0.123	0.025	0.552
23:0	0.241	0.013	0.235	0.023	0.279	0.035	0.247	0.018	0.736
22:4*n*-6	3.21^a^	0.144	2.52^b^	0.185	2.98^ab^	0.183	2.73^ab^	0.163	0.031
22:5*n*-6	0.804	0.099	0.532	0.187	0.719	0.312	0.940	0.061	0.192
22:5*n*-3	0.280^ab^	0.064	0.340^a^	0.019	0.410^a^	0.036	0.160^b^	0.008	0.000
24:0	1.11	0.141	0.893	0.115	0.991	0.163	0.999	0.099	0.705
22:6*n*-3	11.4	0.601	13.0	0.707	10.9	0.857	13.1	0.724	0.084
24:1*n*-9	1.43	0.252	1.14	0.162	1.27	0.211	1.25	0.167	0.800
Others	1.88^a^	0.119	1.58^b^	0.084	2.13^a^	0.158	2.66^a^	0.166	0.000
Partial sums and ratios									
Total SFA	42.6	0.891	42.6	1.40	43.5	1.40	41.2	1.01	0.634
Total MUFA	23.0	1.07	23.2	0.545	22.8	0.311	22.9	0.814	0.886
Total PUFA	32.5	0.871	32.6	1.09	31.6	1.34	33.2	1.03	0.787
Total *n*-3	17.0	0.536	19.0	0.835	17.2	0.816	18.8	0.697	0.084
Total *n*-6	14.5^a^	0.436	12.8^b^	0.339	13.5^ab^	0.570	13.3^ab^	0.332	0.018
*n*-3/*n*-6	1.18^c^	0.035	1.48^a^	0.048	1.27^bc^	0.036	1.42^ab^	0.025	0.000
Major phospholipid classes									
NPL	31.1^b^	0.934	34.0^ab^	1.37	36.0^a^	0.861	33.6^ab^	2.73	0.023
PE	33.4	0.732	33.4	0.956	31.9	0.621	34.3	1.55	0.288
PS	21.9	1.21	20.5	2.03	20.5	0.658	18.3	1.89	0.468
PC	13.6^a^	0.141	12.1^ab^	0.706	11.6^b^	0.523	13.9^ab^	0.776	0.016

*n* = 8 per group. ND, not detected. NPL, non-polar lipids; PE, phosphatidylethanolamine; PS, phosphatidylserine; PC, phosphatidylcholine. Total SFA = 14:0 + 16:0*anteiso* + 16:0 + 18:0 + 19:0 + 20:0 + 22:0 + 23:0 + 24:0; Total MUFA = 16:1*n*-9 + 16:1*n*-7 + 18:1*n*-9 + 18:1*n*-7 + 20:1*n*-9 + 20:1*n*-7 + 22:1*n*-11 + 22:1*n*-9 + 24:1*n*-9; Total PUFA = 16:3*n*-4 + 16:3*n*-3 + 16:4*n*-3 + 18:2*n*-6 + 18:4*n*-3 + 20:2*n*-6 + 20:4*n*-6 + 20:5*n*-3 + 22:4*n*-6 + 22:5*n*-6 + 22:5*n*-3 + 22:6*n*-3; Total *n*-3 = 16:3*n*-3 + 16:4*n*-3 + 18:4*n*-3 + 20:5*n*-3 + 22:5*n*-3 + 22:6*n*-3; Total *n*-6 = 18:2*n*-6 + 20:2*n*-6 + 20:4*n*-6 + 22:4*n*-6 + 22:5*n*-6. ^a,b,c^ Means in the same row with different superscripts are statistically different (PDIFF adjust Tukey*, P* < 0.05)

The major phospholipids classes in brain are also shown in Table 6. In comparison to Milk Fat, Nanno fed rats had increased non-polar lipids (NPL) but decreased phosphatidylcholine (PC) (*P <* 0.05). No additional changes were found for phosphatidylethanolamine (PE) and phosphatidylserine (PS) fractions across dietary groups (*P* > 0.05).

### Behaviour, serotonin and catecholamines

The FST outcomes are presented in Table 7 along with serotonin and catecholamines levels in plasma. Rats fed Nanno and Schyzo diets spent more time floating than rats fed Fish Oil (*P <* 0.05). In turn, Nanno had more time immobile than Schyzo (*P <* 0.05). Climbing and swimming were unchanged (*P* < 0.05) by diet. Adrenalin was increased in Schyzo relative to Milk Fat (*P* < 0.05). Fish Oil fed rats had higher dopamine levels than Nanno (*P* < 0.05). Serotonin and noradrenalin presented similar values across dietary treatments (*P* < 0.05).

**Table 7:** FST outcomes, serotonin and catecholamines in plasma

	Milk Fat		Fish Oil		Nanno		Schyzo		Significance
	Mean	SE	Mean	SE	Mean	SE	Mean	SE	
Climbing (s)	59.5	9.75	75.5	10.1	57.0	14.0	68.3	14.2	0.628
Swimming (s)	119	20.1	125	24.3	82.1	13.3	135	18.7	0.110
Floating (s)	58.4^ab^	8.37	37.7^b^	5.82	78.9^a^	14.2	67.8^a^	6.41	0.006
Immobile (s)	67.7^ab^	19.1	58.8^ab^	21.3	76.0^a^	13.5	24.8^b^	6.36	0.006
Total Movement (s)	179	21.7	200	26.1	150	22.7	208	10.3	0.137
Total Immobile (s)	126	21.4	104	25.8	155	22.6	96.7	9.96	0.125
Serotonin (μg/l)	76.3	32.8	21.4	9.73	49.9	13.9	114	41.1	0.061
Noradrenalin (ng/l)	1257	89.1	1377	128	1442	152	1806	184	0.082
Adrenalin (ng/l)	1826^b^	402	3098^ab^	561	4117^ab^	1296	4889^a^	904	0.016
Dopamine (ng/l)	78.9^ab^	18.4	115^a^	23.5	43.1^b^	10.5	58.9^ab^	14.4	0.047

*n* = 8 per group. ^a,b,c^ Means in the same row with different superscripts are statistically different (PDIFF adjust Tukey, *P* < 0.05)

### Neuromodulation transcriptional profile

**Fig. 1:**
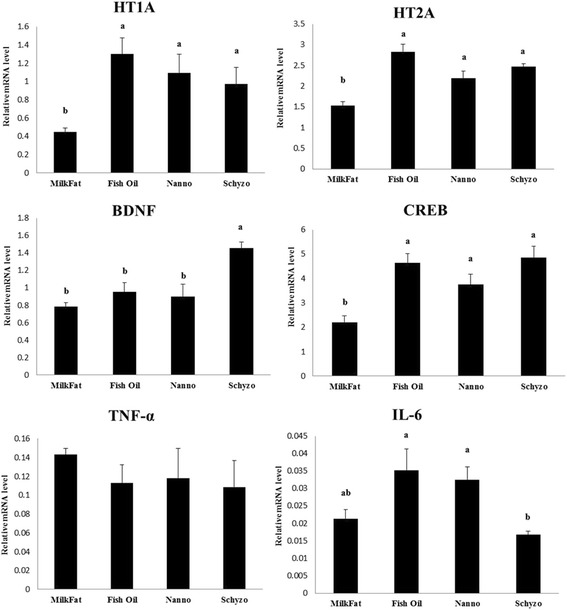
Effect of dietary treatments on the relative expression levels of serotonin 5-HT1A receptor (HT1A), serotonin 5-HT2A receptor (HT2A), brain-derived neurotrophic factor (BDNF), cAMP responsive element binding protein (CREB), tumour necrosis factor-alpha (TNF-α) and interleukin-6 (IL-6) in the hippocampus. Means ± standard error (SE). Means with different letters are significantly different (PDIFF adjust Tukey, *P* < 0.05)

The transcriptional profile of HT1A, HT2A, BDNF, CREB, TNF-α and IL-6 in the hippocampus of rats fed Milk Fat, Fish Oil, Nanno and Schyzo diets are shown in Fig. 1. A similar pattern of variations was found for HT1A, HT2A and CREB being these genes consistently downregulated in Milk Fat in comparison to *n*-3 LCPUFA diets (*P* < 0.05). BDNF gene was upregulated by Schyzo diet (*P* < 0.05). The gene expression levels of IL-6 were equally higher in Fish Oil and Nanno fed rats, and lower in Schyzo (*P* < 0.05). mRNA levels of TNF-α were similar across dietary groups (*P* > 0.05). Moreover, mRNA expression levels of HT1A, HT2A and CREB were moderately (0.7 ≥ *r* ≥ 0.3) positively correlated with *n*-3/*n*-6 PUFA ratio in erythrocytes (*r* = 0.532, *P* = 0.002; *r* = 0.673, *P* < 0.001; *r* = 0.539, *P* = 0.001, respectively).

## Discussion

Facing the premise that EPA and DHA are a safe and inexpensive link to a healthier long life devoid of neurological disturbances, this investigation is of utmost importance to promote sustainability of marine lipid resources and to reduce the environmental impact of fishery without compromising human health needs.

The potential of EPA and DHA to reduce cholesterol levels using experimental animal models has been widely reported [32, 33]. Recently, Ramsden et al. [34] demonstrated that reducing serum cholesterol does not translate into a lower risk from coronary heart disease and improved human survival, increasing the debate on this topic. Notwithstanding, the biggest reduction on LDL-cholesterol was observed in Fish Oil, rather than in Nanno or Schyzo comparative to Milk Fat fed rats. This might be related to the fact that those microalgae enriched diets contained only EPA or DHA, instead of the combined form. Moreover, EPA and DHA combined as well as single EPA reduced total lipids in plasma, in opposition to single DHA; hence, the positive effects found might be due to EPA action. TAG values were higher in Milk Fat fed rats in comparison to the others. Once again, this effect derives from the fact that milk fat did not contain EPA or DHA. Glucose was higher in Milk Fat and lower in *n*-3 LCPUFA enriched diets. Although controversial, several studies point to a diabetogenic effect of SFA with respect to PUFA [35, 36]. This seems to be the case here, given the fact that glucose was higher in rats fed only with Milk Fat. Hepatic markers were measured to determine if microalgae (*Nannochloropsis* and *Schizochytrium*) could be toxic. This is a crucial fact because DHA increases the activity of detoxification enzymes in the liver [37], as verified in Fish Oil and Schyzo fed rats for AST and ALT. Even if AST was deviated from the reference values (42.9 ± 10.1 U/l) [38], *n*-3 LCPUFA enriched diets did not affect liver and renal function, as indicated by the normal values of urea and creatinine. The small variations observed among dietary groups are believed to have no pathophysiological impact.

Regarding cytokines, the non-variation of systemic TNF-α and gene expression levels in the hippocampus is somewhat surprising as high levels of this pro-inflammatory marker were expected in Milk Fat fed rats. Moreover, anti-inflammatory effects have been assigned to dietary EPA and DHA [39, 40]. As far as IL-6 concerns, contradictory findings were observed for this pleiotropic circulating cytokine secreted by many cell types [41]: on the one hand, Schyzo diet increased IL-6 concentration in plasma; on the other hand, IL-6 was down-regulated in the hippocampus of the same dietary group, making it very hard to draw definite conclusions, even if these determinations were performed in different tissues. IL-6 has been reported to increase hepatic synthesis of cholesterol [41] which possibly explains the high levels of cholesterol found in Schyzo fed rats relative to the other *n*-3 LCPUFA diets.

The FA profile in faeces was assessed to determine FA loss, compared to the amount provided by diet. The fatty acid content was higher in Fish Oil and lower in Nanno which means that Nanno fed rats had a higher absorption of FA in the organism. *Nannochloropsis* is indeed efficiently incorporated into the blood, liver and brain lipids of rats [32]. EPA was found higher in Nanno and residual in Fish Oil, which concurs with diet composition. The same explanation applies to Milk Fat or Schyzo with no traces of EPA in faeces. As expected, DHA was not detected in faeces of any dietary group because DHA is highly retained by the body [42, 43].

Liver is the key organ in lipid and lipoprotein metabolism [44]. It is very sensitive to dietary FA variations [44], as observed here. Indeed, *n*-6 PUFA content declined while *n*-3 PUFA generally increased with substitution of fat richer in *n*-3 LCPUFA, thus leading to a marked improvement in *n*-3/*n*-6 ratio. These changes may be mainly attributed to LA reduction and to EPA and DHA overall increase and corroborate previous studies in which liver FA profile displayed approximately 2-fold increase of DHA% after dietary supplementation with DHA and other *n*-3 LCPUFA [44, 45]. Moreover, liver is considered to be the major site for the conversion of ALA into DHA [46]. Our results seem to contradict Barceló-Coblijn and Murphy [47], who reported that dietary ALA is a crucial source of *n*-3 LCPUFA for maintaining these FA tissue contents. In this particular case, it can be argued that ALA content was still insufficient for offsetting the effects of the large amount of milk fat.

The FA profile of erythrocytes, representative of systemic FA bioavailability, showed similar alterations to the liver, although in a lower magnitude. PUFA levels were higher than MUFA, which was expected since PUFA was lower in faeces, indicating a better absorption of these FA by the organism. Within PUFA, *n*-6 was found higher over *n*-3, which is also logical because all diets had higher *n*-6 relative to *n*-3. ARA concentrations were elevated in rats fed high levels of LA, likely as a result of higher ARA synthesis rates [48]. EPA, although found in residual levels (less than 1%), was higher in Fish Oil than in Nanno fed rats and this result contradicts diet composition. The reason for this finding is that EPA was more converted to 22:5*n*-3, a downstream product of EPA in Nanno fed rats. DHA was found higher in Schyzo and lower in Nanno which was expected because Nanno diet had no DHA. The residual levels found might be due to the biosynthesis of DHA from the *n*-3 precursor (ALA) [49] or by EPA in the Nanno group [50]. The same conversion might also have happened in Milk Fat group. Taking into account the highest percentage of *n*-3 PUFA and EPA, and also a high percentage of DHA in erythrocytes, Fish Oil is proven the best dietary source for systemic incorporation of these FA in the organism.

On a *per*-weight basis, brain is the tissue richest in lipids [51]. It is the most conservative organ in terms of DHA uptake [43, 44] where it composes ~10% of total FA [52]. DHA is essential for maintaining normal brain structure, function and metabolism and its concentration depends on dietary DHA content as well as liver synthesis from its shorter chain nutritionally essential PUFA precursor ALA [49]. In fact, DHA synthesis directly in brain tissues is very low [53]. Herein, the non-variation of DHA reinforces the notion that DHA is rapidly absorbed and retained in brain cell membranes [42]. We speculate that the levels found might have been incorporated prior to the administration of DHA, through diet [54]. Any ability to detect changes in EPA in the brain is unexpected. EPA is rapidly and extensively β-oxidised upon entry into the brain [55]. Anyway, we found that rats fed Fish Oil had the highest % of EPA, followed by Nanno and Schyzo groups. These data are in accordance with EPA levels found in erythrocytes and faeces, in which Fish Oil group presented the highest and lowest % of EPA, respectively. The undetected EPA in Milk Fat fed rats correlates well with diet composition.

FA composition of the two major phospholipids classes, PE and PC, was found to be specific: PC contains mostly SFA and 18:1 fatty acids, while PE is rich in PUFA [56]. Changes in SFA/PUFA ratio are likely to influence cellular function, which could impair neurophysiological performances [57, 58]. Even if we failed to characterise the FA composition in brain phospholipid classes, our data suggest a compensatory mechanism of reduced PC fraction while increasing NPL (the majority of which is composed of cholesterol) between Nanno and Milk Fat dietary groups. The lipid class-dependent nature of these variations reflects generally differences in intake and metabolism [59]. Lamaziere et al. [51] reported that the provision of fish oil to rats did not modify PS, in accordance to our data, but increased the proportion of DHA-containing PC. The PE fraction, in which DHA accounts for around 25% (wt%) of total FA [60] was similarly altered by *n*-3 PUFA administration [51], in contrast to our findings.

With respect to FST parameters, it would be expected higher immobile and floating times in Milk Fat (without *n*-3 LCPUFA supplementation) fed rats because these behaviours are recognised as passive and non-active indicators, respectively [26]. Regarding immobility, Nanno fed rats spent more time immobile than Schyzo. DHA has been referred as a possible nutrient for retarding depression and anxiety, supporting the lower immobility time found in Schyzo. Notwithstanding, the immobility time found in Milk Fat was lower than expected and not consistent with the hypothesis initially proposed or with other studies [5, 61]. The lack of positive behaviour in Nanno fed rats can be explained by the high levels of EPA found in faeces. Regarding floating time, Nanno and Schyzo were similar to each other, but higher than Fish Oil. Once more, this possibly indicates that single EPA or DHA are not as beneficial as EPA + DHA, in agreement with observations by Mozaffarian and Wu [62] on cardiovascular health. The only setback is that floating time was also identical between Milk Fat and Fish Oil. In fact, Milk Fat behavioural outcomes were similar to other dietary groups. The presence of DHA in brain of rats fed Milk Fat, in identical amounts to those of *n*-3 LCPUFA enriched diets, can help to explain this similarity.

Adrenalin is a stress hormone [63] whose levels seem to decrease with fish oil administration [64, 65]. A tendency for reduced adrenalin was observed in Fish Oil fed rats relative to Nanno and Schyzo, in accordance with literature [64, 65]. However, Milk Fat group had the lowest adrenalin levels but this diet did not contain EPA or DHA. Moreover, its levels are similar to those found in both Fish Oil and Nanno, but different from Schyzo, leading us to the assumption that neither EPA nor DHA affect adrenalin system, with EPA having a less predominant role, contradicting other reports. On the topics of catecholamines regulation, low levels of dopamine have been associated with PUFA deficiency [65–67]. Higher levels of dopamine were found in Fish Oil group whereas lower were found in Nanno. Therefore, it can be assumed that EPA alone has a smaller impact on regulating dopaminergic system, than combined with DHA.

HT1A and HT2A are related to neural function. Levant et al. [68] have shown that rats with low levels of DHA in brain have less serotonin (5-HT) and higher hippocampal density of 5-HT1A. Serotonin in plasma was found similar across dietary groups although a tendency for high levels were found in Schyzo fed rats, which concurs with diet composition. The transcriptional profile of HT1A and HT2A was consistently upregulated by EPA, DHA or both FA, rather than only by DHA. Accordingly, more research on this subject is required. The variation of CREB expression is positive, given its role in learning and memory, brain traumas recovery and stress [69]. Although not consensual [68], it corroborates the literature pointing towards the normalization of CREB by *n*-3 LCPUFA enriched diets. Also, HT1A, HT2A and CREB mRNA levels in hippocampus were found positively correlated with *n*-3/*n*-6 PUFA in erythrocytes. This ratio is considered a biomarker of systemic inflammation [70, 71] consolidating, once again, the neuroprotective effects of EPA and DHA. CREB is also a transcription factor contributing for BDNF regulation [72, 73]. This later gene was found powerfully expressed by individual DHA.

## Conclusions

The underlying hypothesis of this study was that EPA and DHA would have distinct neurobiological effects, if ingested singly or combined, using alternative marine lipid sources as *n*-3 LCPUFA rich microalgae in Wistar rats. FST revealed the potential benefit of fish oil (EPA + DHA) compared to microalgae oils (EPA or DHA singly). These results could be ascribed to high concentrations of systemic dopamine and *n*-3 LCPUFA incorporation in liver and erythrocytes, suggesting that fish oil is a better dietary source for FA deposition in the organism. Moreover, each tissue, liver, erythrocytes and brain, showed a particular FA profile with specific traits. Another positive impact of a diet rich in both EPA and DHA was the mitigation of plasma metabolites unbalance, in particular through reduction of total lipids and LDL-C/HDL-C ratio. In turn, EPA provided by *Nannochloropsis* microalga had similar results, although with a lower magnitude as EPA and DHA combined, in opposition to DHA provided by *Schizochytrium* microalga, allowing us to conclude that the protective effects on plasma lipid profile might be due, to a large extent, to EPA action. Apart from the positive variation of BDNF mRNA levels by DHA alone, the combination of EPA and DHA can provide protection against reduced plasticity and impaired learning ability by upregulating HT1A, HT2A and CREB genes in the hippocampus. Taken together, a diet enriched in EPA + DHA form seems more adequate for health promotion, in various critical domains of neurophysiology and lipid metabolism, which can benefit, in the long run, neuronal structure and function.

## Abbreviations

γ-GT: gamma glutamyl transferase
AA: arachidonic acid
ALA: α-linolenic acid
ALP: alkaline phosphatase
ALT: alanine aminotransferase
AST: aspartate aminotransferase
BDNF: brain-derived neurotrophic factor
CREB: cAMP responsive element binding protein
DHA: docosahexaenoic acid
EPA: eicosapentaenoic acid
FA: fatty acid
FAME: fatty acid methyl esters
FST: forced swimming test
HT1A: serotonin 5-HT1A receptor
HT2A: serotonin 5-HT2A receptor
IL-6: interleukin-6
LA: linoleic acid
*n-*3 LCPUFA: *n-*3 long chain polyunsaturated fatty acids
NPL: non-polar lipids
PC: phosphatidylcholine
PE: phosphatidylethanolamine
PS: phosphatidylserine
TAG: triacylglycerols
TNF-α: tumour necrosis factor alpha
